# Supplementary material for: Identification and functional analysis of non-coding regulatory small RNA FenSr3 in Bacillus amyloliquefaciens LPB-18
Source: PeerJ. 2023 May 15;11:e15236. doi: 10.7717/peerj.15236 (PMC10194069; doi:10.7717/peerj.15236)
Supplement: Supplemental Information 4 [file peerj-11-15236-s004.zip › KO/CK-vs-T1_map/map04136.html]

KEGG PATHWAY: Autophagy - other - Reference pathway


|  |  |
| --- | --- |
| **Autophagy - other - Reference pathway** |  |

[
Pathway menu
| Organism menu
| Pathway entry
| Show description
| User data mapping
]

Autophagy is a degradative pathway for the removal of cytoplasmic materials in eukaryotic cells, and is characterized by the formation of a double-membrane structure called the autophagosome, either in a housekeeping capacity or during stress and senescence. The process of autophagy could be divided into several stages: induction, vesicle nucleation, elongation and closure, and fusion and digestion. Most essential autophagic machineries are conserved throughout eukaryotes (see map04140 for animals and map04138 for fungi). This map is for other eukaryotes including plants and protists, where autophagy related genes (ATGs) play similar roles in the life cycle. However, autophagy has been relatively less studied in lower eukaryotes.

|  |  |  |
| --- | --- | --- |
| Reference pathway | 184% 150% 122% 100% 82% 67% 55% | 图片下载 |
